# Minimum requirements in emergency kits for bailout strategies in TAVR complications

**DOI:** 10.1111/jocs.16380

**Published:** 2022-03-06

**Authors:** Marco Gennari, Gilbert H. L. Tang, Maurizio Taramasso, Giulio Russo, Philipp K. Haager, Marco Barbanti, Ana Paula Tagliari, Francesco Maisano

**Affiliations:** ^1^ Department of Cardiovascular Surgery IRCCS Centro Cardiologico Monzino Milan Italy; ^2^ Department of Cardiovascular Surgery Mount Sinai Health System New York City New York USA; ^3^ Department of Cardiovascular Surgery Klinik Hirslanden Zürich Switzerland; ^4^ Department of Invasive Cardiology Fondazione Policlinico Universitario A. Gemelli, IRCSS Roma Italy; ^5^ Department of Cardiology Kantonsspital St. Gallen St. Gallen Switzerland; ^6^ Division of Cardiology, Policlinico‐Vittorio Emanuele Hospital, C.A.S.T University of Catania Catania Italy; ^7^ Department of Cardiac Surgery, Postgraduate Program in Health Sciences: Cardiology and Cardiovascular Sciences Faculdade de Medicina da Universidade Federal do Rio Grande do Sul Porto Alegre Brazil; ^8^ Heart Valve Center IRCCS San Raffaele Hospital Milan Segrate Italy

**Keywords:** bailout, coronary occlusion, TAVR, vascular complications

## Abstract

**Introduction:**

The proportion of patients with symptomatic severe aortic stenosis treated by transcatheter aortic valve replacement (TAVR) is increasing, driven by favorable outcomes from randomized trials and current valve guidelines recommendations. Despite device and technique improvements having reduced procedural morbidities, complications during or immediately after TAVR still carries significant mortality risk.

**Methods:**

We propose a check‐list of essential items to anticipate potentially life‐threatening complications in TAVR.

**Results:**

Purpose of this review is to discuss the most common life‐threatening complications during TAVR from a troubleshooting perspective, depicting the minimum required equipment needed in emergency situations.

**Conclusions:**

Prevention of complications remains the most important strategy to optimize outcomes in TAVR procedures. Each specialized Center should institute and make easily accessible standardized emergency kits for the most common life‐threatening conditions during TAVR that should be readily available in the cath‐lab or hybrid operating room.

**Abbreviations**
COBT: cross‐over balloon technique
CPB: cardio‐pulmonary bypass
EACTS: European Association for Cardio‐Thoracic Surgery
ESC: European Society of Cardiology
EVAR: endovascular aortic repair
PPM: permanent pacemaker implantation
SAVR: surgical aortic valve replacement
SOV: sinus of valsalva
STJ: sino‐tubular junction
TAVR: transcatheter aortic valve replacement
THV: transcatheter heart valve
ViV: valve‐in‐valve

## 1 INTRODUCTION

Transcatheter aortic valve replacement (TAVR) has transformed the way to treat patients suffering from severe symptomatic aortic stenosis (AS) and has become the preferred treatment. With evidence from multiple randomized trials, the latest joint European Society of Cardiology/European Association for Cardio‐Thoracic Surgery and American College of Cardiology (ACC)/American Heart Association (AHA) guidelines for the management of valvular heart disease,^1^ have expanded current TAVR indications.

Even though technological improvements and increased operator experience have led to a decrease in major periprocedural complications,^2^, ^3^, ^4^, ^5^ the need of bailout strategies to manage potentially life‐threatening complications remains.^6^ Currently, there are no specific international guidelines focused on the management of the most common life‐threatening TAVR complications. Nevertheless, a prompt and standardized approach to each individual type of complications along with preassembled emergency kits seem to be useful. Based on our experience of over more than 1000 TAVR cases at our respective institutions, we have developed a strategy of having different preassembled emergency kits readily available off the shelf to manage a wide range of complications.

## 2 METHODS

We reviewed the main periprocedural TAVR complications and described the minimum requirements in emergency kits (Table 1) and the subsequent bailout strategies in a pragmatic easy‐to‐understand manner.

**Table 1:** Basic emergency kits for managing the most common complications during transfemoral TAVR

Basic emergency kit for high‐degree/complete AV blocks
6F Venous introducer sheath
0.032”–0.035” stiff guidewire
External pulse generator
Multipolar electrophysiologic catheter
Black subdermal electrode needles
Connectors, electrodes, alligator clips
Basic emergency kit for pericardiocentesis
16–18 Gauge needle (minimum 15 cm of length)
20–50 ml syringe
0.035” J‐tip stiff guidewire
large‐bore Pigtail‐shaped Catheter (e.g., 8.3F)
Luer‐Lock taps systems
Graduated draining bag with connectors
Basic emergency kit for transcatheter valve retrieval
6F arterial sheath
0.035” extra stiff guidewire
6F AL‐1, AL‐2 guiding catheters
Snare‐based catheters systems of different sizes Goose neck snare Multi‐snare Ensnare
Non‐snare‐based retrieval systems Graspers Biopsy forceps
Compliant Balloon Catheters of different sizes
Noncompliant Balloon Catheters of different sizes
Basic emergency kit for vascular complications and aortic rupture
12F–14F arterial Sheath
0.018” guidewire
0.018” hydrophilic Guidewire
5F Internal Mammary Artery Catheter
8, 10, 12 mm Peripheral Balloons
8, 10, 12 mm covered self‐expanding stents
0.035” Extra Stiff guidewire
RELIANT™ Stent Graft Balloon Catheter (*Medtronic, MN, USA*)
Y‐Connector
Basic emergency kit for coronary occlusion
6F AL1 guiding catheter
6F JR1 guiding catheter
0.014” Coronary wires (including hydrophilic and hi‐torque)
Universal BMW
Torquer
Coronary balloons of different sizes
Coronary stents of different sizes
Y‐Connector
Basic emergency kit for cardio‐pulmonary bypass institution
0.035” Back‐up Meier™ wire (*Boston Scientific, MA, USA*), or similar
17F arterial cannula
24F venous cannula
Vascular dilators
Silk sutures
Connectors

Abbreviations: AV, atrioventricular; TAVR, transcatheter aortic valve replacement.

## 3 RESULTS

### 3.1 High degree/complete atrioventricular (AV) blocks

Due to the proximity of the AV node to the aortic valvular complex, postprocedural high‐degree or complete AV block requiring permanent pacemaker implantation are one of the most common complications after TAVR, ranging from 2% to 51% (average 17%).^7^

The diagnosis is easily achieved by intraprocedural electrocardiogram monitoring; most frequently the electric disturbances appear after the valve deployment; in a small percentage the blocks are delayed, hours or even days after the procedure.

A bailout strategy for managing high‐degree blocks is crucial, especially in the setting of minimalist TAVR approach with pacing through the left ventricular wire. In the latter case, especially when implanting self‐expanding devices that require guidewire maneuvering to and from the left ventricle, it is important assuring that major heart rhythm disturbances have not developed before removing the wire.

The bailout strategy involves prompt transvenous pacing. The venous access and 6F sheath introduction are achieved by a classic Seldinger technique. The sheath harbors the passage of a multipolar catheter to be connected to an external pulse generator. The use of a guiding catheter can be useful to navigate the right ventricle in case of super‐acute cavo‐atrial boundary. A VVI setting at the appropriate sensing ad energy output is recommended.

If the patient remains temporary pacemaker‐dependent, assure proper fixation of the electrodes to the skin and consider to exchange for an atraumatic pacing catheter, especially in the context of very elderly patients at risk of right ventricular perforation. Check the settings and battery level of the pulse generator. Always have a spare battery to the pulse generator in the patient's room!

### 3.2 Cardiac tamponade/pericardial effusion

**Figure 1:**
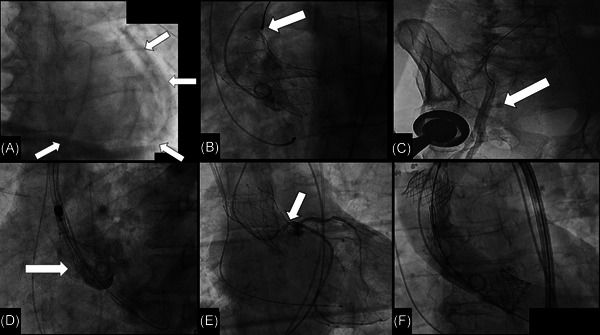
(A) Emergent pericardiocentesis for acute hemopericardium; the draining Pigtail catheter is inserted within the pericardial sac after fluoroscopic confirmation of the correct needle position by contrast dye injection (*arrows*). (B) Snaring of a self‐expanding valve implanted too low (too ventricular). (C) Infrainguinal arteriogram showing dissection and extravasation of contrast medium at the end of a TAVR procedure. (D) Ascending aorta dissection (*arrow*) detected at the post‐deployment angiogram. (E) Left main stenosis (arrow) after transcatheter aortic valve deployment causing ST elevation on electrocardiogram. (F) Self‐expanding prosthesis implantation subsequent ascending aorta embolization. Final result after second implantation of an Edwards Sapien 3 balloon‐expandable valve

One of the most fearful complications after TAVR is hemopericardium (Figure 1A); any time of the procedure could be complicated by pericardial effusion due to left or right ventricular perforation, annular rupture, or injury from temporary pacemaker insertion or withdrawal.^8^

The key for the success is to quickly evacuate the pericardial space due to cardiac tamponade and possibly facilitate the clotting process. The diagnosis is made by clinical and echocardiographic evaluation (new onset and increasing pericardial effusion on transoesophageal or transthoracic echocardiogram with progressive hypotension unresponsive to fluid administration).

Two tools are mandatory in this context: a pericardiocentesis set and a cell saver.

### 3.3 Pericardiocentesis set

The emergent pericardial drainage could be performed under transesophageal, transthoracic and/or fluoroscopic guidance. Full reversal of the anticoagulation state by the infusion of protamine should be pursued on a case‐by‐case basis since it can risk thrombus formation.

In the normal TAVR scenario a fluoroscopic‐guided procedure is often performed using the antero‐posterior and lateral projections to remark the pericardial shadow.^9^ A mix saline‐iodinate medium (e.g., 70%/30%, respectively) may help in verify the correct intra‐pericardial access under fluoroscopy. The technique involves the puncture at the left sub‐xiphoid, at 15 degrees to the skin to overpass the diaphragm border. The advancement should be slow and echo and/or fluoro‐guided, oriented towards the patients’ left shoulder. Once reached the intrapericardial space the blood should be quickly aspired to reverse the tamponade status. A standard “J‐tip 0.035” guidewire is thereafter inserted under fluoroscopic guidance and the draining 8Fr Pigtail inserted over the wire and connected to a graduate sac. An overall hemodynamic and echocardiographic assessment is recommended at this stage; in case of clinical improvement and absence of recurrence we recommend close observation at an intensive care unit. The patient should be check continuously. The Pigtail catheter can be removed if the daily output is less than 50 ml, and after echocardiographic confirmation showing absence of pericardial effusion.

In case of a more severe bleeding, a direct blood reinfusion through the femoral introducer sheath could be performed after heparin readministration.

In case of intractable bleeding a prompt sub‐xiphoid surgical drainage or full sternotomy is mandatory. A good rule of thumb is to observe the amount of bleeding in the first 5 min. If the bleeding is more than 300 ml per minute after 2–3 min, or if the patient remains severely unstable, then conversion to surgery is mandatory. In our practice, we deliver a full dose of heparin before sternotomy to establish peripheral cardiopulmonary bypass (femoral‐femoral), with a standard set including pump, oxygenator, reservoir and multiple suction lines, because in most occasions the lesion leading to massive bleeding requires full mechanical support and the possibility to reinfuse the aspirated blood drainage. On some occasions the lesion can be repaired on the beating heart, otherwise a cardioplegic arrest is needed to facilitate a complex repair (annular rupture, aortic dissection, large ventricular injury). In case of myocardial lesions from guidewire perforation we recommend using large sutures with large needles and large Teflon pledgets to mattress the ventricular wall, since the bleeding spot appearing on the surface is often larger than it appears.

#### 3.3.1 Cell saver

Cell savers are instruments that collect and gather blood lost during surgery or complex interventional procedures. The red blood cells (RBCs) are washed with normal saline and concentrated to make an approximate 225 ml unit with a hematocrit of approximately 55% that can be stored or immediately reinfused to the patient. This tool is effective to promptly restore the normotonic volemia.

The system works by the connection of the Pigtail catheter draining the hemopericardium to an outflow‐line to the central venous access.

The cell saver is useful given its quick setup, reducing the delay in seeking allogenic blood units. Cell saver could be also useful in cases of complex surgical correction.

### 3.4 Device embolization

The VARC‐3 3 defines^10^ valve migration as the dislodgement of the transcatheter valve after initial correct positioning; the valve prosthesis moves upwards or downwards, within the aortic annulus from its initial position, with or without consequences.

Valve embolization refers to the valve prosthesis movement during or after the deployment such that it loses its contact with the aortic annulus.

Device embolization is currently a relatively rare complication of TAVR (0.92% in the largest multicenter TRAVEL registry),^11^ mainly due to device improvements and more accurate preprocedural sizing and intraprocedural deployment. However, device migration or embolization can still occur either in the upward (aortic) or downward (left ventricular outflow tract) direction requiring urgent management. Aortic embolization remains the most common direction (70%–80%) of device dislodgement.

More than 80% of valve migration is immediately peri‐procedural, 15% within the first hour and only 3% thereafter. The diagnosis is made from fluoroscopy and echocardiogram.

In most cases the preferred management is to address the issue via catheter‐based approaches.^12^

Three are the main bailout strategies to be pursuit:

1. Repositioning the transcatheter heart valve (THV)
2. Implanting a second valve or valve‐in‐valve (ViV)
3. Conversion to surgery

In case of THV embolization (Figure 1F) rule #1 is to keep the wire across the prosthesis, particularly in cases of balloon‐expandable valve, that can tumble and obstruct the blood flow.

Here are our proposed management steps:

(a) where to place the embolized valve? Snare (Figure 1B) and pull it back to the ascending aorta or descending aorta (for self‐expanding THV, mainly). Secure it with an uncovered stent (in case the valve has been tilted against the direction of antegrade blood flow).
(b) in case you decide (or you need) to leave the embolized prosthesis in the ascending aorta, retrieve it distal to the the Sinus of Valsalva (SoV) to avoid coronary occlusion after second THV deployment and, before advancing the second valve, secure it with a snare while you are advancing the delivery system carrying the second THV, to prevent any migration toward the SoV.
  It is crucial to achieve an additional arterial accessfor the snare to be able to keep the snared valve in position while advancing the second one
(c) choose the most appropriate second THV according to the patient's anatomy to reduce the risk of coronary occlusion
(d) if the wire position was not lost, proceed with delivering the second device.^13^

If wire position was lost, the embolized valve must be re‐crossed, using a Pigtail catheter to avoid crossing and getting stuck within stent struts.

Once a central position of the guidewire is achieved the native aortic valve must be recrossed and, holding the embolized valve in place with a snare, the second valve can be implanted as usual.

In case one cannot grasp the valve frame with the snare it is useful to get a Terumo glidewire (*Terumo, Shibuya, Tokyo, Japan*) across one strut of the THV, and try to catch the wire with the snare.

In certain scenarios, surgical conversion can be considered according to the patient and family's wishes and the patient's surgical risk. This is usually necessary in case of ventricular embolization.

### 3.5 Vascular complications and aortic rupture

Peripheral vascular complications remain at 5%–8% in transfemoral (TF) TAVR, despite the relative low‐profile sheaths of currently available devices, more accurate preprocedural planning and micro‐puncture access along with ultrasound or fluoroscopic‐guided access and preclosure techniques.

Pelvic vascular injuries such as bleedings (Figure 1C) and dissections causing flow‐limiting lesions, or pseudoaneurysm are the most common vascular complications of TF‐TAVR.

On the other hand, injuries of the great vessels (i.e., thoracic or abdominal aorta, subclavian artery) are rarer but can be catastrophic, needing surgical or endovascular bailout strategies but carries a poor prognosis.^14^

In case of using radial artery as secondary arterial access to perform bailout strategies,^15^ it is crucial before starting to know the equipment available on site, since the dimensions of the sheaths as well as the guidewires, catheters and stents lengths may not reach the target peripheral vessel!

### 3.6 Bailout strategies for peripheral vascular injuries

The diagnosis of ileo‐femoral dissection, disruption or pseudoaneurysm is suspected clinically and confirmed by fluoroscopy, typically by digital subtraction angiogram.

Cross‐Over Balloon Technique from the contralateral common femoral artery could be performed over a “0.018” or “0.035” guidewire and with a 5Fr IMA (Internal mammary artery), UF (Universal flush) or JR (Judkins right) catheter. An appropriately sized balloon catheter inflated proximally to the lesion allows achieving temporary hemostasis while appropriate final treatment is planned.

In small, localized dissection a balloon dilatation for 15 min can be sufficient to achieve vascular sealing. Otherwise, a peripheral stent is required. Consider partial reversal of the heparinization effect to facilitate hemostasis. Peripheral self‐expanding covered stent‐grafts may be needed in case of vessel lacerations. Make sure to apply an oversizing of at least 15% to prevent endoleaks. A preventive crossover wire is a good option in case of complex femoral anatomies with high risk of periprocedural lesions.

In case of very challenging cross‐over anatomies (tortuosity, calcification and steep bifurcation angle), the alternative is to perform a ipsilateral distal access puncturing the superficial femoral artery to place the stent retrogradely. In this case it is fundamental to puncture the distal access at a sufficient distance to allow easy and effective maneuvering.

Involving a vascular surgeon early or pre‐emptively may avoid these potential complications and can be life‐saving.

### 3.7 Bailout strategies for thoraco‐abdominal aorta injuries

This rare by devastating complication is suspected clinically by the rapid onset of profound hypotension, generally soon after valve deployment, or device's introducer insertion or removal.

At fluoroscopy, a huge extravasation of contrast medium or an evident intimal flap may appear. The first goal is to avoid exsanguination. An occlusive balloon inflated just proximal to the rupture site may help buy time to provide further interventional treatments such endovascular aortic repair (EVAR) with available endografts.

Open surgery can also be an option, particularly dissection of the ascending aorta that may occur either after wire and catheters manipulations or after valve deployment (Figure 1D).

### 3.8 Acute coronary occlusion

Acute coronary obstruction after TAVR is a rare but potentially devasting complication, carrying as much as 50% of mortality.^16^ The best way to prevent this life‐threatening complication is an accurate preoperative evaluation of coronary obstruction risk.^17^

In recent years, the development of the interventional technique called BASILICA^18^ to intentionally split native or bioprosthetic aortic leaflets has led the achievement of good outcomes even when a high risk of occlusion is expected. Nonetheless, the technique is quite challenging and requires a proper learning curve. Also, commissural alignment of the THV is also necessary to avoid the commissural post obstructing the split portion of the leaflet after a successful BASILICA.

It is well‐established that ViV procedures, especially those involving stentless bioprosthesis or stented bioprosthesis with externally mounted leaflets, low‐coronary ostia (<10 mm from the aortic basal plane) and a shallow SoV and sino‐tubular junction (STJ) present a four to sixfold increased risk of coronary obstruction.^19^ Nevertheless, in native aortic valve anatomy this complication may occur in the presence of a tall and bulky leaflet facing the coronary ostium (Figure 1E).

An accurate preprocedural evaluation, including a planning for adjunctive techniques for coronary protection such as prophylactic chimney snorkel^20^ have led to a reduction of this complication. Once occurred, a prompt interventional management is mandatory to improve outcomes.

### 3.9 Emergent cardio‐pulmonary bypass (CPB) institution

Life‐threatening TAVR complications causing refractory cardiogenic or hemorrhagic shock can be managed by expeditious institution of CPB as a bridge‐to‐decision with temporary cardio‐respiratory support.^21^ Both hybrid operating suites and catheterization laboratories performing structural heart procedures must be equipped to quickly setup extracorporeal membrane oxygenation (ECMO) support. An on‐site perfusionist on call is mandatory to help operate the mechanical circulatory support equipment. Some Institutions have a full CPB machine running on stand‐by, which can be used for support or surgical conversion.

We do not use ECMO systems unless there is a need for postoperative mechanical circulatory support. The fastest way to institute the mechanical support is using the primary femoral vascular access site as the default entry for cannula placement.

The arterial cannula should be advanced to the common iliac artery, not too deep to reduce the risk of ipsilateral hypogastric ischemia (check at fluoroscopy!). Alternatively, if the large‐bore TAVR introducer sheath is already in place in the femoral artery, inserting a venous cannula (with multiple holes as arterial outflow) directly through the sheath with the cannula tip positioned at the descending aorta reduces the risk of vascular complication by avoiding sheath exchanges, while providing expeditious CPB with adequate flow to stabilize the patient to guide further management decisions. The venous cannula consists of two stages and should be advanced through the cavo‐atrial junction to maximize the blood drainage under fluoroscopic and transesophageal echocardiographic guidance. The de‐airing process while connecting the cannulae to the CPB machine is crucial for the arterial outflow; this can be achieved by a gentle flushing of sterile saline while connecting the tubings to the CPB circuit.

Before the patient being transferred, securing the cannula by silk stitches on the skin and proper dressing is mandatory to avoid cannula dislodgment or removal.

## 4 COMMENT

Although serious TAVR complications are quite rare in the current era, they still carry significant morbidity and mortality. Prevention remains the most important strategy but when a complication occurs, it is crucial to make a prompt diagnosis and treatment plan. In the absence of international guidelines, each operator normally adopts “personal” protocols that may be not be standardized, leading some delay in the management even within the same Institution.

## 5 CONCLUSION

Each specialized Center should institute and make easily accessible standardized emergency kits for the most common life‐threatening conditions during TAVR that should be readily available in the cath‐lab or hybrid operation room. It may improve the readiness of the team to treat the emergency situation and may help improve the outcome.

## CONFLICTS OF INTEREST

Gilbert H.L. Tang: physician proctor for Medtronic; is a consultant for Medtronic, Abbott, and NeoChord; and is an advisory board member for Abbott and JenaValve. Maurizio Taramasso: consultant for Abbott Vascular, Boston Scientific, 4tech, CoreMedic. Speaker fees from Edwards Lifesciences. Francesco Maisano: Grant and/or Research Support from Abbott, Medtronic, Edwards Lifesciences, Biotronik, Boston Scientific Corporation, NVT, Terumo Consulting fees, Honoraria from Abbott, Medtronic, Edwards Lifesciences, Xeltis, Cardiovalve. Royalty Income/IP Rights: Edwards Lifesciences. Shareholder (including share options) of Cardiogard, Magenta, SwissVortex, Transseptalsolutions, Occlufit, 4Tech, Perifect. All other authors have reported that they have no relationships relevant to the contents of this paper to disclose.

## AUTHOR CONTRIBUTIONS

**Marco Gennari and Gilbert H.L. Tang**: conceptualization and writing. **Maurizio Taramasso, Marco Barbanti, and Francesco Maisano**: supervision. **Giulio Russo, Ana Paula Tagliari, and Philipp Haager**: data curation.
